# The prevalence of genome replacement in unisexual salamanders of the genus *Ambystoma *(Amphibia, Caudata) revealed by nuclear gene genealogy

**DOI:** 10.1186/1471-2148-8-158

**Published:** 2008-05-22

**Authors:** Ke Bi, James P Bogart, Jinzhong Fu

**Affiliations:** 1 Department of Integrative Biology, University of Guelph, Guelph, Ontario N1G 2W1, Canada

## Abstract

**Background:**

Unisexual salamanders of the genus *Ambystoma *exemplify the most ancient lineage of unisexual vertebrates and demonstrate an extremely flexible reproductive system. Unisexual *Ambystoma *interact with and incorporate genomes from two to four sexual species (*A. laterale*, *A. jeffersonianum*,*A. texanum*, and *A. tigrinum*), to generate more than 20 genome compositions or biotypes. Unisexual ploidy levels range from diploid to pentaploid, but all contain at least one *A. laterale *(L) genome. Replacement of nuclear genomes might be responsible for the evolutionary longevity of unisexual *Ambystoma *but direct evidence for the prevalence of genome replacement in natural populations is absent. Two major puzzling questions have remained unanswered over the last few decades: 1) is genome replacement a common reproductive method in various unisexual populations and, 2) is there an ancient "L" genome that persists in various unisexual genome compositions.

**Results:**

We examined 194 unisexual and 89 *A. laterale *specimens from 97 localities throughout their range and constructed a genealogy of the "L" genomes using a nuclear DNA marker (L-G1C12) to answer the above questions. Six L-G1C12 haplotypes (A-F) were shared by individuals in various *A. laterale *and unisexual populations. The general geographical distribution of the haplotypes in unisexual populations conformed to those found in *A. laterale*, indicating that "L" genomes in unisexuals are obtained from sympatric or nearby populations of *A. laterale*.

**Conclusion:**

Our data demonstrate that genome replacement frequently occurs in unisexual *Ambystoma *across their range, and support previous speculations that genome replacement is an important reproductive mechanism that can enhance their evolutionary longevity. Our results show that there is no ancient "L" genome in the unisexual lineages, and no particular "L" genome is favored in any unisexual individual. The presence of an "L" genome in all unisexuals implies that it is important to the maintenance of unisexuals. Nuclear gene genealogy is a powerful tool to examine the historical interaction between sperm-dependent unisexuals and their sexual sperm donors. This methodology could be applicable to many other unisexual lineages to improve our understanding of their reproduction and their ability to persist.

## Background

The evolution of sexuality versus asexuality has been the focus of considerable attention for decades. Although sexuality is ubiquitous in animals, unisexual reproduction has independently evolved in various unrelated lineages [1-3]. The existence of ancient unisexuals, regardless of their rarity, poses a dilemma for evolutionary biologists because a lack of genetic recombination should, theoretically, cause unisexuals to be short-lived [4-10]. From a single hybrid origin, unisexual salamanders in the genus *Ambystoma *have survived for 2.4–3.9 million years [11], representing the most ancient known unisexual vertebrate lineage [12,13]. Unisexual *Ambystoma *have a unique, while extremely flexible, reproductive system which is described as kleptogenesis [11]. Female kleptogens usually produce unreduced eggs through premeiotic endomitosis [14], and sperm from sympatric sexual males is required to activate the eggs without fertilization (termed gynogenesis). But, a sperm nucleus can be incorporated to elevate the ploidy (termed ploidy elevation) or to replace one of the nuclear genomes in a developing offspring (termed genome replacement) [1]. Unisexual individuals interact with and "steal" chromosomes from two to four sympatric sexual species (*A. laterale *(LL), *A. jeffersonianum *(JJ), *A. texanum *(TT), and *A. tigrinum *(TiTi)), and more than 20 distinct diploid, triploid, tetraploid and pentaploid genome compositions are known to exist [15]. Among these reproductive mechanisms, genome replacement may be responsible for the apparent evolutionary longevity of unisexual *Ambystoma *[12,16,17].

Although the evolutionary significance of genome replacement in unisexual *Ambystoma *is acknowledged, empirical evidence for its prevalence in natural populations is absent. An ancient genome replacement event was proposed using a phylogenetic approach [15]. All the unisexual *Ambystoma*, irrespective of their genome compositions, share an almost identical mitochondrial genome that is distinctly different from any of the four "parental" species, excluding all of them as candidates for a maternal ancestor. The sequence of unisexual *mt*DNA is most closely related to that in specimens of *A. barbouri *("BB") from Kentucky, suggesting that *A. barbouri *and all unisexual salamanders share a close common maternal ancestor. However, the "B" nuclear genome was completely replaced, but left its mitochondrial genome behind [11]. Nevertheless, the existence of genome replacement was questioned by Spolsky et al. [13,16]. They found no evidence supporting genome replacement in a few *A. texanum*-dependent LJJ populations in central Indiana and northeastern Illinois so they believed that genome replacement in unisexual *Ambystoma *is very uncommon in nature. But, they acknowledged the potential importance of genome replacement for long-term survival of unisexual *Ambystoma*. Previous studies using isozyme electrophoresis, DNA microsatellite and cytogenetic analyses had been carried out to try to document the actual occurrence of genome replacement in a few unisexual populations [11,18-24]. All these studies present discrete evidence that demonstrate the possible rare occurrence of genome replacement but the methods used were insufficient to document genome replacement across a large number of unisexual populations. As a consequence, none of those studies provide conclusive empirical evidence about the frequency or commonality of genome replacement in unisexual *Ambystoma*. To better document to what extent genome replacement takes place in this old lineage, nuclear gene genealogy between sexual species and their corresponding genomic contributions in various unisexual populations across the range could be the key to understand this process.

Despite the presence of genome replacement, all known unisexual *Ambystoma*, irrespective of their genome compositions, contain at least one "L" genome, even in those populations where no *A. laterale *have ever been discovered [25]. The conservation of an "L" genome in every unisexual is a mystery. It was hypothesized that hybridization between a female *A. barbouri *and a male *A. laterale *gave rise to an ancestral genome composition "BL" that initiated a unisexual linage. Subsequently, early in the history of unisexual linage, the "B" genome was replaced by genomes from other sperm donors, but the ancient paternal "L" genome may have been maintained [15]. Recent evidence contradicted this hypothesis [11]. DNA microsatellite loci demonstrated that, in one population from southern Ontario, no common genome is transmitted in a clonal or hemiclonal fashion in unisexuals, and that there is not a single "L" genome that could be considered ancestral or frozen in all unisexuals [11]. Nevertheless, additional empirical evidence is required for many other unisexual populations with respect to whether there exists a particular "L" genome that is always favored and trapped in unisexual individuals, and is somehow protected from genome replacement.

Directly examining the genealogical relationships of the "L" genomes within the unisexual and sexual lineages provides a rapid way to answer the following two fundamentally important questions. First, is genome replacement a common or sporadic event during the evolution of unisexual *Ambystoma *lineages? If it is rare, we would expect that most "L" genomes in unisexual lineages would be more closely related to each other than to respective sympatric *A. laterale*. Second, is there a particular *"*L" genome that is frozen in unisexual populations across their distributional range? If there is, we would expect that one "L" genome found in individual unisexuals from distant populations would group together and have a more basal position on the "L" genome tree. In this study, we chose a highly variable DNA fragment from a nuclear gene intron as a marker, designed specific primers that only amplified the fragment in the "L" genome in various unisexual genome compositions, and constructed a genealogy for "L" genomes in unisexuals and *A. laterale*.

## Results

### Haplotypes and general distribution

**Figure 1:**
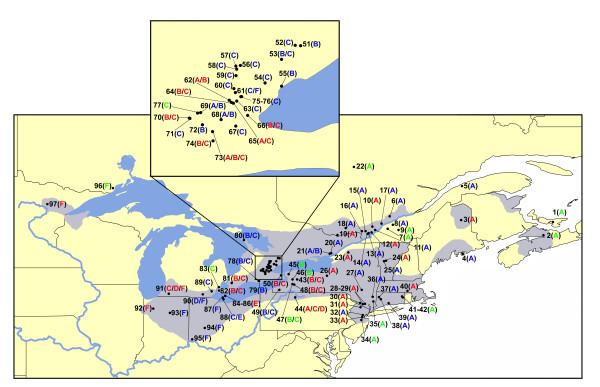
**Distribution of 97 sampling sites and geographic distribution of L-G1C12 haplotypes in unisexuals and *Ambystoma laterale *in northeastern North America **(see Additional file 1 for description of locations and sample sizes). Numbers represent sampling sites where we collected specimens for this study. The shaded grey area shows the current known range of unisexual *Ambystoma*. Small map on the top corresponds to the boxed area on the large map. A-F represent six L-G1C12 haplotypes. Blue and green letters indicate populations where we only sampled unisexuals or *A. laterale*, respectively. Red letters indicate populations where both unisexual and *A. laterale *were found and they shared the same haplotypes. Multiple letters divided by slashes represent populations where more than one haplotype was found.

**Figure 2:**
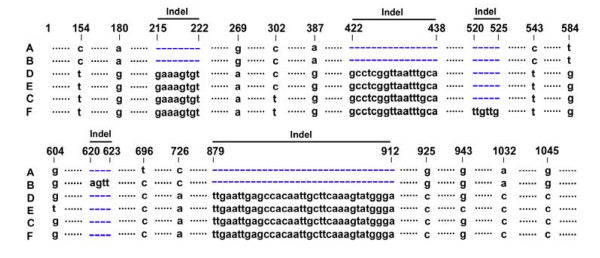
**The variable sites of sequences of six haplotypes (A-F)**. Five indels and 14 informative variable nucleotide sites were identified. The deleted sties are highlighted as blue dashes.

We successfully amplified the DNA marker (G1C12) in all unisexual and *A. laterale *individuals and in one specimen from each of the outgroup species (*A. jeffersonianum*, *A. texanum*, and *A. tigrinum*) (GenBank accession numbers EU647505–EU647513). In this study we considered each haploid "L" genome as a unit. *Ambystoma laterale *contains two haploid "L" genomes. In unisexuals, for example, an LJJ contains one "L" genome, an LLJ contains two "L" genomes and an LLLJ contains three. Six distinct L-G1C12 haplotypes (A-F) were found in both unisexuals and *A. laterale *(Additional file 1, Figure 1). No haplotype was unique to either unisexual or *A. laterale *specimens. Among these six haplotypes, 14 informative variable nucleotide sites and five indels (insertions/deletions) were observed (Figure 2). The size of five indels found in the ingroup samples varied, which resulted in the different length of these six haplotypes. The geographical distribution of each haplotype is mapped on Figure 1. Haplotye A (993 bp) was most widespread with a frequency of 44% in unisexual individuals. This haplotype was found in all eastern populations and a few individuals in our collection of central populations. Haplotype B (997 bp, freq. 18%) was only found in populations from central areas, and was especially prevalent in southwestern Ontario and western New York. Haplotype C (1052 bp, freq. 26%) was a common haplotype that was shared by unisexuals and *A. laterale *in the central-western region. Haplotype F (1058 bp, freq. 7%) was mostly distributed in the western edge of the unisexual distribution. Haplotypes D and E were less common and distributed in central areas. Haplotype D (1052 bp, freq. 2%) was found in one population in Pennsylvania (site 44) and two populations in Michigan (sites 90–91). Haplotype E (1052 bp, freq. 3%) was found only in individuals from the Lake Eire islands (sites 84–86, 88). In general, haplotypes A and F were found in a homozygous condition in most east and west *A. laterale *and unisexual populations respectively. Haplotypes B, C, D and E dominated central area, where many of the *A. laterale *and unisexual individuals were heterozygous for two different haplotypes.

### Genealogy of "L" genomes in unisexuals and in *A. laterale*

**Figure 3:**
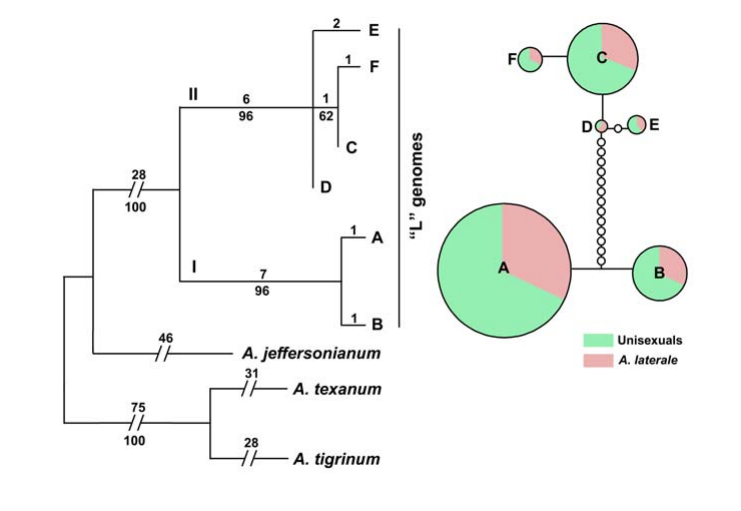
**Maximum parsimony tree (left) and TCS analysis (right) for six L-G1C12 haplotypes recovered from populations of *Ambystoma laterale *and unisexuals**. For the MP tree, the outgroup species are *A. jeffersonianum*,*A. texanum*, and *A. tigrinum*. Taxa are haplotypes (A-F). Numbers above the branches represents numbers of mutations, and below depicts bootstrap values greater than 50. There is a distinct genetic break between clade I [A, B] and clade II [C, D, E, F]. For the TCS haplotype network, haplotypes A-F are represented by circles whose areas are proportional to the frequencies of the particular haplotype. The relative frequencies of unisexuals vs *A. laterale *partitioned in each particular haplotype are shown by different colors (green and pink respectively). Small and empty circles represent intermediate haplotypes that are not present in the samples but are necessary to link all observed haplotypes to the network. All haplotypes are separated from the nearest haplotype by one nucleotide difference. A distinct genetic break (13 transitional steps) between [A, B] and [C, D, E, F] is identified by TCS.

The topology from both maximum parsimony and maximum likelihood analyses were identical. We only present the former in Figure 3. Compared with the outgroup species, haplotypes of "L" genomes from unisexuals and *A. laterale *formed a distinct clade with high nodal support. It is noteworthy that the consistency index (CI) of the ingroups is 1.00, indicating that all pieces of information support the hypothesis. The most obvious feature of our tree is the well-supported division of L-G1C12 haplotypes into two major clades. Clade I contains haplotypes A and B and clade II contains C, D, E, and F. A deep genetic divergence (10 single mutational changes and three gaps) was found between these two major clades. In clade I, A and B differed by one single mutation and one gap. In clade II, C to F differed by three single mutations and one gap (Figure 2).

The haplotype relationship of L-G1C12 was further analyzed by TCS (Figure 3). The network revealed a distinct deep divergence (13 mutational steps) between two groups that corresponded to haplotypes [A, B], and [C, D, E, F] respectively, which was consistent with our phylogenetic tree.

It is evident from Additional file 1 and Figure 1, that the general distribution of L-G1C12 haplotypes in both unisexual and *A. laterale *specimens was congruent. Unisexual and *A. laterale *individuals from the same populations share the same haplotypes. For those populations where only unisexual specimens were sampled or where unisexuals did not use *A. laterale *as a sperm donor, such as LJJ populations, the distributional pattern of haplotypes in unisexuals were found to be consistent with those in *A. laterale *from their nearby locations.

## Discussion

### The prevalence of genome replacement in unisexual *Ambystoma*

Genome replacement is common in the unisexual lineage throughout evolutionary time. Our results clearly demonstrate that the geographical distribution of six L-G1C12 haplotypes shows concordance in various unisexual and *A. laterale *populations. We find no evidence to support that "L" genomes in unisexuals have evolved independently and diverged from those in sympatric *A. laterale*. Nuclear gene genealogy successfully tracks the signature of genome replacement in natural populations and offers strong support that genome replacement is a common reproductive mode that has been widely used by various unisexual *Ambystoma *populations across their entire distribution. The historical and contemporary frequency of genome replacement in unisexual *Ambystoma *is, however, difficult to quantify. Nevertheless, the six haplotypes found in unisexual populations, including homozygous and various heterozygous combinations, provide indisputable evidence of major replacement events involving *A. laterale *in the unisexual linage.

The general low genetic divergence of "L" genomes in unisexuals and *A. laterale *is not surprising given their recent range expansion [12]. During the last glacial maximum of the Wisconsin (ca. 18,000 years ago), most of the present-day range of *A. laterale *and unisexuals was covered by the Laurentide Ice Sheet [26,27]. We sequenced 17 different nuclear loci on the "L" genomes but only G1C12 provided sufficient variation among "L" genomes from various *A. laterale *and unisexual populations to test our hypothesis. In their phylogeographical study of *A. laterale*, only four parsimony informative sites were found in the non-protein-coding region of *mt*DNA that resulted in clear segregation of *A. laterale *into two distinct groups (East-central and West) [27]. Our data, that used L-G1C12 as the nuclear DNA marker, also demonstrate a very similar East/West dichotomy which strengthens our confidence that our marker does track evolution in *A. laterale*.

Although all unisexuals possess at least one *A. laterale *genome, the success of unisexual individuals in expanding their range requires their dependence on sexual sperm donors and the habitat where they were initially formed. Our results show that many of the unisexuals that do not use *A. laterale *as a current sperm donor have L-G1C12 haplotype allocations that are most similar to those found in *A. laterale *from geographically nearby localities. It indicates historical genome replacement events when they could have interacted, followed by subsequent dispersal and colonization of unisexuals.

### No preference of a particular "L" genome in unisexual *Ambystoma*

Based on mitochondrial DNA, unisexual *Ambystoma *have a single origin [11] and no recurrent hybridization between any two of the four genomic donors has been responsible for subsequent unisexual lineages. It was postulated that *A. laterale *may have been the paternal ancestor of this old lineage based on the observation that all the unisexual genome compositions contain at least one "L" genome [15]. Our results clearly demonstrate that no ancient or particular *A. laterale *genome is preferentially preserved in any unisexual *Ambystoma*, rejecting the hypothesis that unisexual individuals may preferentially maintain one particular "L" genome. Any and all of the "L" genomes in various unisexual genome compositions seem to be replaceable. Other nuclear markers, such as highly variable DNA microsatellite loci, were also used to demonstrate that there is not a consistent "L" genome that stays in one population in southern Ontario [11]. Genome replacement would explain the variation between individuals from discrete egg masses and the presence of the same microsatellite alleles in unisexual individuals and sympatric sperm donors [11]. The existence of an "L" genome in every known unisexual (>23 genome compositions) is more than just a coincidence and begs an explanation. The disjunction of evolutionary histories of the nuclear and mitochondrial genomes of the unisexual *Ambystoma *still poses a very interesting question regarding why an "L" genome is so important to unisexuals. One possible explanation might be related to the significance of the interaction between nuclear and mitochondrial genomes to an organism [28].

The conservation of an "L" nuclear genome may also hold a key to our understanding of sex determination in unisexual *Ambystoma*. If all ambystomatids have a ZZ/ZW sex determination system [29], the replacement of a maternal "B" genome should have resulted in unisexual genomes devoid of a W chromosome. Subsequent genome replacements and additions can only contribute new Z chromosomes to developing embryos so the offspring should theoretically consist of males. To explain this situation, it was speculated that the preservation of "femaleness" in unisexual *Ambystoma *could be accomplished by an intergenomic translocation that transferred the W-sex gene from the ancient "B" chromosome to an "L" chromosome [30]. However, our results provide no support for this hypothesis because no ancient "L" genome, that could contain any *A. barbouri *genome-derived sex determining locus, is maintained in unisexuals.

### Genome replacement as an important reproductive mechanism that can provide longevity to unisexual lineages

During the past few decades, many researchers have argued about evolutionary challenges of unisexuals by questioning their persistence [31]. For example, three dominant hypotheses ('Red Queen', 'Müller's ratchet' and the 'mutation-load-reduction theory') have been proposed and revisited in recent years to account for why unisexuality is difficult [7]. Generally, many believe that unisexuality would be "a ticket to swift extinction" [4] because unisexuals lack genetic recombination so they can not generate the variation to cope with changing environments, to purge deleterious mutations and gene combinations and to repair damaged DNA [2,6]. However, the detection of ancient unisexual lineages in invertebrates and vertebrates is peculiar so understanding how these organisms sustain viability without sex can potentially help us better understand why sex is important [32]. To persist, many unisexual lineages are capable of incorporating unusual reproductive modes that circumvent normal sexual reproduction allowing recombination to occur [11,33]. Genome replacement and ploidy elevation is especially important for sperm-dependent unisexuals to incorporate additional genetic material by transmission of genomes or genes from a sexual host therefore providing a source of adaptive flexibility [17,34]. Genome replacement has been demonstrated in hybridogenetic unisexual fishes (*Poeciliopsis *complex [35,36], the Iberian minnow *Leuciscus alburnoides *[2,37]), frogs (*Rana esculenta *complex [38]) and kleptogenetic unisexual *Ambystoma *(present study). Genome replacement explains how lineages may compensate for the disadvantages of unisexuality and all these groups demonstrate genetic and ecological flexibility as well as evolutionary potential so they may not be condemned to an evolutionary "dead-end". These unisexuals may not be the only organisms that enjoy such evolutionary flexibility. It was posited that most ancient unisexual lineages could show some degree of paternal leakage or genome replacement [17]. Theoretically, even a minimal amount of sex may be very crucial for unisexuals to bypass a fatal accumulation of deleterious mutations [39] and it could be particularly significant for lineages to maintain long-term survival [17]. Recent evidence using modern molecular techniques support the fact that few (if any) ancient unisexuals reproduce completely asexually [9]. The present discovery of commonality of genome replacement in unisexual *Ambystoma *suggest that it has probably been an important factor in the evolution of this complex, and the unisexual *Ambystoma *likely have much more dynamic genomic histories than previously believed. The broader implications for genome replacement, especially the consequences of novel genome compositions to sperm-dependent unisexual vertebrates, remain to be further explored.

## Conclusion

Unisexual kleptogens steal sperm from suitable sperm donating species. Most commonly, the sperm is not incorporated and the embryonic development is gynogenetic. The fusion of the sperm nucleus with an unreduced oocyte results in ploidy elevation and this phenomenon is commonly observed in many unisexual populations. Genome replacement is more difficult to observe without suitable genetic markers because the replaced genome may be genetically equivalent to those that are not replaced. In the unisexual *Ambystoma*, genome replacement and ploidy elevation are considered important evolutionary mechanisms to compensate for the disadvantages of unisexual reproduction. Genome replacement is an effective method to remove deleterious mutations that may accumulate in a specific genome and/or to acquire advantageous genes. We have found that replacement of the *A. laterale *genome in unisexual populations has commonly occurred over the range of unisexual individuals that are sympatric with *A. laterale*. Our data emphasize the importance of kleptogenesis during the long evolutionary history of the unisexual lineage. Likewise, other genomic constitutes in unisexual individuals most likely followed a similar "refreshment" process repeatedly through their sympatric association with other sexual sperm donors. If such replacement of genomes, followed by novel genetic expression and epigenetic regulations, occurred many times over historical time, these events would increase the evolutionary flexibility to enable the long-term sustainability of the unisexual lineage. Genome replacement may also be common in other unisexual animals. Gene genealogy using genome-specific markers is an effective method to investigate the general distribution and commonality of genome replacement in unisexual lineages.

## Methods

### Specimen sampling and lab protocols

DNA was extracted from 194 unisexual and 89 *A. laterale *specimens that were collected from 97 geographically isolated localities (Additional file 1, Figure 1). Most samples included larva, juvenile or adult tissues that had been used in previous isozyme investigations [11,40,41] and kept frozen at -80°C. Some more recently collected samples were also obtained in Ontario, Michigan and Ohio. The genome composition for each specimen was identified using isozyme analysis, karyotyping, microsatellite DNA loci and/or flow cytometry. Nomenclature of unisexual *Ambystoma *was described by Lowcock et al. [42]. For example, an "LJJ" represents a unisexual triploid salamander possessing one chromosome set of *A. laterale *and two sets of *A. jeffersonianum *chromosomes.

Total genomic DNA was isolated using Promega Wizard Genomic DNA Purification Kits. To look for candidate nuclear DNA markers, we tested 17 different nuclear loci. However, only one of these examined loci yielded sufficient variation among "L" genomes from different unisexual and *A. laterale *populations. The nuclear DNA marker we chose was selected based on a nuclear expressed sequence tag-based (EST) locus from *A. tigrinum*, named as G1C12 [43]. This locus was identified to flank an intron boundary in a gene displaying similarity to the *Homo sapiens *myosin regulatory light chain MRCL2 sequence. We used the original primers G1-C12.5.1 and G1-C12.3.2 [43] to amplify this sequence in all five sexual species (LL, JJ, TiTi, TT, and BB) that are involved in unisexual *Ambystoma *reproduction and got amplifications in all but *A. barbouri *(BB). G1-C12.5.1 and G1-C12.3.2 were also used to identify the sequences of major haplotypes in various *A. laterale *populations. By aligning the sequences from the four sexual specimens we found a few indels, which allowed us to redesign primers that targeted these regions in order to obtain species-specific sequences. We modified the original primers as L-G1C12F1 (5'-TGGCCTCACTTGGTAAGTGTC-3') and L-G1C12R1 (5'-CACGCTTATCCACAAAGTCTC-3'). The reverse primer was located on a region where the other three species (JJ, TiTi, and TT) contain a large insertion so this primer can only anneal with sequences from *A. laterale*. Primers were repeatedly tested on all five sexual species and our results showed, that with a high annealing temperature of 58°C, they specifically amplified *A. laterale *(L-G1C12) and not the other four sexual species. These modified primers were then used to amplify this marker from "L" genomes in various unisexual genomic combinations. Standard polymerase chain reaction (PCR) amplifications were performed using an MJ Research PTC-200 Peltier Thermal Cycler. PCR amplification of L-G1C12 was carried out in 25 μl reactions containing 1× PCR buffer, 0.1 mM dNTP, 0.15 U of Taq DNA polymerase, 1μl of each primer (10 pmol/μl), and *A. laterale *genomic DNA. The PCR program included 1 min initial denaturation at 94°C, 35 cycles of 94°C/58°C/70°C for 30 s/40 s/1 min followed with a 5 min extension at 72°C. PCR products were verified on 1% agarose gels and purified using Qiagen QIAquick PCR purification kits. Clean products were then used as templates for sequencing PCR using both forward and reverse primers and Big Dye terminator sequencing reaction, according to the manufacture's protocol. Sequences were resolved on an ABI 3730 DNA Analyzer, verified by Sequencher (version 4.5) and aligned using ClustalX [44]. Heterozygous sequences that contained ambiguous aligned regions or multiple heterozygous nucleotide sites were identified and then cloned using PGEM T-easy vector system II (Promega) to verify the sequence of each sequence in heterozygous individuals. We also amplified the same gene sequence using original primers G1-C12.5.1 and G1-C12.3.2 from *A. jeffersonianum*, *A. texanum*, and *A. tigrinum*, which served as the outgroups for phylogenetic construction.

### Phylogenetic method

A gene genealogy using maximum parsimony and maximum likelihood was constructed using PAUP* (version 4.0b10) [45]. Each unique nuclear haplotype was treated as a taxon and each nucleotide site was treated as a character. The four nucleotides (ACGT) were the character states. Gaps were treated as fifth character states. For gaps that were longer than one single nucleotide site, all but one nucleotide site was excluded. This eliminated the character weighting effect. For example, an insertion of a fragment with 20 nucleotides would be considered as one evolutionary event, rather than 20 events. All characters were weighted equally and unordered. A heuristic search method via tree-bisection-reconnection branch swapping with 1000 random step-wise addition replicates was used. To generate the maximum likelihood tree, the evolutionary model that best fit the observed data, based on the likelihood ratio test, was calculated using Modeltest (Version 3.6) [46]. The output for both hierarchical likelihood ratio and Akaike information criterion tests produced the best scores for our data. A 1000 bootstrap replicates was conducted with the preferred model in PAUP* 4.0b10. Because maximum likelihood analysis does not allow gaps as fifth character states, each gap was converted to one single transversional change. A haplotype network was built based on the principle of statistical parsimony using the computer program TCS 1.21 [47,48]. The treatment of gaps was the same as was used in the maximum parsimony analysis.

## Authors' contributions

KB detailed the experimental design and performed most of the lab work, data analyses and manuscript preparation. JPB and JF conceived and directed the study. All authors contributed equally to this work in discussing research strategy and development. All authors read and approved the final manuscript.

## Supplementary Material

Additional file 1 Sampling locations, sample sizes (n) and L-G1C12 haplotype distributions for *Ambystoma laterale *and unisexual individuals from sites in northeast North America. The table shows the detailed allocation of L-G1C12 haplotypes in both *Ambystoma laterale *and unisexual specimens across the entire distributional range (see Figure 1 for the geographical distribution of sites and haplotypes). Click here for file
