# Supplementary material for: The prevalence of genome replacement in unisexual salamanders of the genus Ambystoma (Amphibia, Caudata) revealed by nuclear gene genealogy
Source: BMC Evol Biol. 2008 May 22;8:158. doi: 10.1186/1471-2148-8-158 (PMC2413238; doi:10.1186/1471-2148-8-158)
Supplement: Additional file 1 — Sampling locations, sample sizes (n) and L-G1C12 haplotype distributions for Ambystoma laterale and unisexual individuals from sites in northeast North America. The table shows the detailed allocation of L-G1C12 haplotypes in both Ambystoma laterale and unisexual specimens across the entire distributional range (see Figure 1 for the geographical distribution of sites and haplotypes). [file 1471-2148-8-158-S1.pdf]

Additional file 1

Supplementary Table - Sampling locations, sample sizes (n) and L- G1C12 haplotype distributions for *Ambystoma laterale* and unisexual individuals from sites in northeast North America (see Figure 1 for the geographical distribution of sites and haplotypes).

Site	Location	n		L-G1C12 haplotypes[1]	
		A. laterale	Unisexual	*A. laterale*	Unisexual
1	Prince Edward Island	1	n/f [2]	A(2)	-
2	Cumberland County, Nova Scotia	2	n/a [3]	A(4)	-
3	Aroostook County, ME	1	1 (LLJ)	A(2)	A(2)
4	Hancock County, ME	n/a	1 (LLJ)	-	A(2)
5	Ste. Blandine, Quebec	n/a	2 (LLJ)	-	A(4)
6	Bastican, Quebec	n/a	2 (LLJ)	-	A(4)
7	St. Jude, Quebec	1	n/a	A(2)	-
8	Yamaska, Quebec	n/a	1 (LJ)	-	A(1)
9	Drummond, Quebec	2	n/a	A(4)	-
10	St. Jacques, Quebec	1	4 (LLJ)	A(2)	A(8)
11	Repentigny, Quebec	n/a	2 (LLJ)	-	A(4)
12	Bos St. Laurent, Quebec	4	2 (LLJ)	A(8)	A(4)
13	Breckenridge, Quebec	n/a	1 (LLJ)	-	A(2)
14	Anse-a-l'Orme, Quebec	n/a	1 (LLJ)	-	A(2)
15	Mirabel, Quebec	n/a	1 (LLJ)	-	A(2)
16	Lachute, Quebec	n/a	2 (LLJ)	-	A(4)
17	Lanoraie, Quebec	n/a	1 (LLJ)	-	A(2)
18	Ripon, Quebec	n/a	1 (LJ)	-	A(1)

19	Aylmer, Quebec	2	6 (LLJ),1 (LLLJ)	A(4)	A(15)
20	Leeds/ Grenville Counties, Ontario	n/a	1 (LLJ)	-	A(2)
21	Leeds/ Grenville Counties, Ontario	n/a	1 (LLJ)	-	A(1),B(1)
22	Chapais, Quebec	2	n/f	A(4)	-
23	St.Lawrence County, NY	2	1 (LLJ)	A(4)	A(2)
24	Addison County, VT	1	2 (LLJ)	A(2)	A(4)
25	Addison County, VT	n/a	1 (LJ)	-	A(1)
26	Seneca/Wayne Counties, NY	2	2 (LLJ)	A(4)	A(4)
27	Schoharie County, NY	n/a	2 (LJ)	-	A(2)
28	Orange County, NY	1	1 (LLLJ)	A(2)	A(3)
29	Orange County, NY	3	4 (LLJ),1(LLLJ)	A(6)	A(11)
30	Orange County, NY	2	1 (LJ),1(LLJ)	A(4)	A(3)
31	Sussex County, NJ	1	1 (LLJ)	A(2)	A(2)
32	Sussex County, NJ	n/f	1 (LJJ)	-	A(1)
33	Morris County, NJ	2	4 (LLJ),1(LLLJ)	A(4)	A(11)
34	Morris and Sommerset Counties, NJ	2	n/a	A(4)	-
35	Putnam County, NY	1	n/a	A(2)	-
36	Dutchess County, NY	n/f	1 (LJJ)	-	A(1)
37	Litchfield County, CT	n/f	1 (LJJJ)	-	A(1)
38	Litchfield County, CT	n/a	3 (LLJ)	-	A(6)
39	New Haven County, CT	n/f	2 (LJJ)	-	A(2)
40	Worcester County, MA	2	2 (LLJ)	A(4)	A (4)

41	Windham County, CT	2	n/a	A(4)	-
42	Windham County, CT	1	n/a	A(2)	-
43	Genesee County, NY	2	1 (LLJ)	B(1),C(3)	B(1),C(1)
44	McKean County, PA	4	4 (LLJ)	A(3),C(3),D (2)	A(4),C(3), D(1)
45	Niagara County, NY	3	n/a	B(6)	-
46	Erie County, NY	2	n/a	B(4)	-
47	Erie County, NY	2	n/a	B(3),C(1)	-
48	Erie County, NY	2	2 (LLJ)	B(2),C(2)	B(1),C(3)
49	Chautauqua County, NY	n/a	3 (LLJ)	-	B(5),C(1)
50	Welland County, Ontario	3	2 (LLJ)	B(4),C(2)	B(3),C(1)
51	York County, Ontario	n/a	1 (LLJ)	-	B(2)
52	York County, Ontario	n/f	3 (LJJ)	-	C(3)
53	York County, Ontario	n/f	2 (LJJ)	-	B(1),C(1)
54	Halton County, Ontario	n/f	2 (LJJ)	-	C(2)
55	York County, Ontario	n/f	1 (LJJ)	-	B(1)
56	Peel County, Ontario	n/f	3 (LJJ)	-	C(3)
57	Peel County, Ontario	n/f	2 (LJJ)	-	C(2)
58	Peel County, Ontario	n/f	2 (LJJ)	-	C(2)
59	Halton County, Ontario	n/f	1 (LJJ)	-	C(1)
60	Halton County, Ontario	n/f	1 (LJJ)	-	C(1)
61	Halton County, Ontario	n/f	4 (LJJ),1 (LJJJ)	-	C(4),F(1)
62	Wentworth County, Ontario	3	1 (LLJ)	A(4),B(2)	A(1),B(1)
63	Halton County, Ontario	n/f	1 (LJJJ)	-	C(1)

64	Wentworth County, Ontario	3	2 (LLJ)	B(3),C(3)	B(2),C(2)
65	Wentworth County, Ontario	2	3 (LLJ)	A(2), C(2)	A(3),C(3)
66	Wentworth County, Ontario	1	1 (LLJ)	B(1),C(1)	B(1),C(1)
67	Wentworth County, Ontario	n/f	3 (LJJ)	-	C(3)
68	Wentworth County, Ontario	n/a	1(LLJ)	-	A(1),B(1)
69	Waterloo County, Ontario	n/a	4 (LLJ)	-	A(4),B(4)
70	Waterloo County, Ontario	1	2(LLJ),2(LJJ),1(LJ)	B(1),C(1)	B(6),C(1)
71	Waterloo County, Ontario	n/f	2 (LJ),4 (LJJ)	-	C(6)
72	Waterloo County, Ontario	n/f	3 (LJJ)	-	B(3)
73	Waterloo County, Ontario	5	5 (LLJ),1(LLLJ)	A(2),B(3),C (5)	A(5),B(6),C (2)
74	Brant County, Ontario	1	2 (LLJ),1(LLLJ)	B(1),C(1)	B(3),C(4)
75	Wellington County, Ontario	n/f	1 (LJJ)	-	C(1)
76	Halton County, Ontario	n/f	1 (LJJ)	-	C(1)
77	Waterloo County, Ontario	2	n/a	C(4)	-
78	Haldimand/ Norfolk Counties, Ontario	n/a	3 (LLJ),5 (LJJ)	-	B(6),C(5)
79	Haldimand/ Norfolk Counties, Ontario	n/f	4 (LJJ)	-	B(4)
80	Bruce County, Ontario	n/a	1 (LLJ)	-	B(1),C(1)
81	Lambton County, Ontario	3	4 (LLJ)	B(2),C(4)	B(1),C(7)
82	Essex County, Ontario	1	3 (LLJ)	B(1),C(1)	B(2),C(4)

83	Walpole Is., Essex County, Ontario	1	n/a	C(2)	-
84	Pelee Is., Essex County, Ontario	n/f	3 (LTT)	-	E(3)
85	Pelee Is., Essex County, Ontario	1	1 (LLT)	E(2)	E(2)
86	Pelee Is., Essex County, Ontario	2	n/a	E(4)	-
87	North Bass Is., Ottawa County, OH	n/f	1 (LTT)	-	F(1)
88	Kelleys Is., Erie County, OH	n/f	4 (LTTi)	-	C(1),E(3)
89	Washtenaw County, MI	n/f	2 (LJJ)	-	C(2)
90	Lenawee County, MI	n/a	1 (LLJ)	-	D(1),F(1)
91	Cass County, MI	2	5(LLJ),2(LLLJ),1(LTTi)	C(1), D(2),F(1)	C(9),D(5), F(3)
92	Jasper County, IN	1	1 (LLJ)	F(2)	F(2)
93	Wabash County, IN	n/f	2 (LJJ)	-	F(2)
94	Clark County, OH	n/f	1(LTTi),4(LTJ),2(LTJTi)	-	F(7)
95	Hamilton County, OH	n/f	4 (LJJ)	-	F(4)
96	Thunder Bay Dist., Ontario	2	n/f	F(4)	-
97	Itasca County, MN	2	1 (LLJ)	F(4)	F(2)

[1]A-F represent six haplotypes. Parenthetical letters represent unisexual genome combinations. Parenthetical numbers represent the numbers of haplotypes found in specimens when considering each haploid "L" genome as a unit. For example in site 73, five LLJ and one LLLJ unisexuals were examined and they contain in total 13 haploid "L" genomes. "A(5), B(6), C(2)" means that five of these 13 "L" genomes contain haplotype A, six of these 13 "L" genomes contain haplotype B and two of these 13 "L" genomes contain haplotype C.

[2] n/f: *Ambystoma laterale* or unisexual was never found from the locality.

[3] n/a: *Ambystoma laterale* or unisexual was not sampled from the locality but could be present.
